# Barrier materials for prevention of surgical adhesions: systematic review

**DOI:** 10.1093/bjsopen/zrac075

**Published:** 2022-06-06

**Authors:** Michael Gerard Waldron, Conor Judge, Laura Farina, Aoife O’Shaughnessy, Martin O’Halloran

**Affiliations:** Translational Medical Device Laboratory, National University of Ireland Galway, Galway, Ireland; Translational Medical Device Laboratory, National University of Ireland Galway, Galway, Ireland; Translational Medical Device Laboratory, National University of Ireland Galway, Galway, Ireland; Translational Medical Device Laboratory, National University of Ireland Galway, Galway, Ireland; Translational Medical Device Laboratory, National University of Ireland Galway, Galway, Ireland

## Abstract

**Background:**

Postoperative surgical adhesions constitute a major health burden internationally. A wide range of materials have been evaluated, but despite constructive efforts and the obvious necessity, there remains no specific barrier widely utilized to prevent postoperative adhesion formation. The aim of this study was to highlight and characterize materials used for prevention of postoperative surgical adhesions in both animal and human studies.

**Methods:**

A systematic review was performed of all original research articles presenting data related to the prevention of postoperative adhesions using a barrier agent. All available observational studies and randomized trials using animal models or human participants were included, with no restrictions related to type of surgery. PubMed and Embase databases were searched using key terms from inception to August 2019. Standardized data collection forms were used to extract details for each study and assess desirable characteristics of each barrier and success in animal and/or human studies.

**Results:**

A total of 185 articles were identified for inclusion in the review, with a total of 67 unique adhesion barrier agents (37 natural and 30 synthetic materials). Desirable barrier characteristics of an ideal barrier were identified on review of the literature. Ten barriers achieved the primary outcome of reducing the incidence of postoperative adhesions in animal studies followed with positive outputs in human participants. A further 48 materials had successful results from animal studies, but with no human study performed to date.

**Discussion:**

Multiple barriers showed promise in animal studies, with several progressing to success, and fulfilment of desirable qualities, in human trials. No barrier is currently utilized commonly worldwide, but potential barriers have been identified to reduce the burden of postoperative adhesions and associated sequelae.

## Teaser

In this systematic review, we evaluated barrier materials utilized for the prevention of postoperative adhesions. We assessed 67 adhesions barriers from 185 animal and human studies in the published literature, with each barrier material was assessed based on achieving the primary goal of postoperative adhesion reduction and on the potential ability to achieve a standardized set of desirable characteristics. Multiple barriers showed promise in animal studies, with several progressing to success in and fulfilment of desirable qualities in human trials.

## Introduction

Postoperative adhesions are scar tissue resulting from trauma of the peritoneal surface and have been documented in 79–90 per cent of individuals after open abdominal or pelvic surgery^1–3^. Postoperative adhesions are a leading cause of long-term morbidity following surgery^4–6^, with 27 per cent of patients being re-admitted following abdominal or pelvic surgery for disorders directly related to adhesions within 5 years^6^. Adhesions are associated with significant morbidity including small bowel obstruction (SBO), chronic pain, infertility, and requirement for a repeat procedure^4,7,8^; in addition to the socioeconomic implications^7^, including the significant financial burden with cumulative direct hospital care costs estimated at 2.3 billion dollars in 2011 in the USA alone^9^. Postoperative adhesions are characteristically difficult to treat^4^, with the severity of formed adhesions and rate of iatrogenic bowel injury during adhesiolysis increasing exponentially with each additional operation^7^. Adhesive disease has no specific laboratory or radiological finding that are currently in use in common practice, although cine-MRI has shown potential in providing information related to extent, location, and strength of intra-abdominal adhesions^10^. Prevention or reduction of adhesion formation is a key priority.

A wide range of materials have been evaluated in animal and/or human studies as physical barriers to separate the wound from surrounding tissue in an effort to reduce the rate and severity of postoperative adhesions^9,11,12^; however, despite constructive efforts and the obvious necessity, no specific barrier remains widely utilized in clinical practice to prevent postoperative adhesion formation^13^. Animal studies remain critical to advancing clinical research, as they are biologically similar to humans, susceptible to similar health issues, and have a shorter life cycle allowing testing over a life span and successive generation^14^. However, animal welfare and economic funding must be central to any decision to progress with research. The European Union (EU) Directive 2010/63/EU on the protection of animal welfare was produced to harmonize standards of animal research across the EU^15^. Research using animal models must be carefully designed and relevant, with animal welfare remaining a central concern^14^. Furthermore, a comprehensive listing of studied barriers in animal and human studies is lacking in systematic reviews to date^9,11,12^, prompting the need to investigate the breadth of barriers previously published, including those whose investigation was halted after the animal investigation phase.

The aim of this study was to characterize the strengths and shortcomings of each barrier, comparing tissue adherence (traumatized and oozing tissue); applicability through a laparoscope; safety for the patient; ease of application; postoperative pain; and overall efficacy to reduce the rate and severity of postoperative adhesions. Utilizing the information above, the aim is to identify whether an ideal solution exists or whether a pre-existing barrier shows promise for advancement to further research, and also to assess the pre-existing barriers in terms of their readiness for the market: success in animal study; progression to human study and the outcomes; and product on the market.

## Methods

### Selection criteria

A systematic review was performed according to published guidelines from the Cochrane Collaboration^16^ and is reported according to the PRISMA guidelines^17^. A study protocol (*Appendix S1*) was developed to include original research articles presenting data related to the prevention of postoperative adhesions using a barrier agent. Studies involving physical barrier agents and non-physical barriers were included. Studies of non-resorbable barriers (such as polytetrafluoroethylene), where a further interventional procedure would be necessary for removal, were excluded. All published observational studies and randomized trials were included if they met the following criteria: contained original data, used animal or human participants, or evaluated an adhesion barrier(s) in abdominal and/or pelvic adhesions. No date restrictions were applied and there was no restriction on the type of surgery.

### Search strategy

A systematic search of the literature was performed in two databases (PubMed and Embase). The databases were searched from inception to August 2019. The search was performed using key terms: (Surg*(Title/Abstract)) AND (adhesion*(Title/Abstract)) AND (prevent*(Title/Abstract)) AND (barrier*(Title/Abstract)). Two reviewers (M.W. and C.J.) independently screened titles and abstracts using the Rayyan web application for systematic review screening^18^. Full texts were sourced for relevant articles. Inclusion criteria were assessed independently (M.W. and C.J.), and the final list was agreed by consensus with a third reviewer (L.F.). The reference lists of similar review articles were also screened. The systematic review was performed in accordance with the pre-specified protocol, which was prospectively registered on PROSPERO, the international prospective register of systematic reviews (ID CRD42020125090).

### Data extraction

Three standardized data collection forms for animal and human studies respectively were used (*Appendix S2*). For each study, the title, year of publication, barrier type (natural or synthetic), barrier category (categories were finalized after data extraction), generic and brand name (where applicable), and whether the barrier contained a combination of agents, were extracted. The animal model (such as rat, chicken, or rabbit) for animal studies, and the type of surgery performed (such as abdominal or pelvic) for human studies, were recorded. Reviewers (M.W., C.J., and L.F.) independently extracted data, compared for inconsistencies, and merged into a final data set. Discrepancies were resolved following discussion under supervision of the lead author (M.O.H.).

### Appraisal of studies

Additionally, desirable barrier characteristics (*Appendix S3*) including adherence to traumatized tissue, adherence to oozing tissue, application laparoscopically, safety for the patient, cost-effectiveness, postoperative pain, and ease of application were extracted from full-text articles. Pathway to the market characteristics were extracted as listed in *Appendix S4*. Successful barriers were those where positive outputs have been reported for each of the desirable characteristics in previous literature and potentially successful barriers were those that had positive outcomes but a number of desirable characteristics required further research.

## Results

**Fig. 1:**
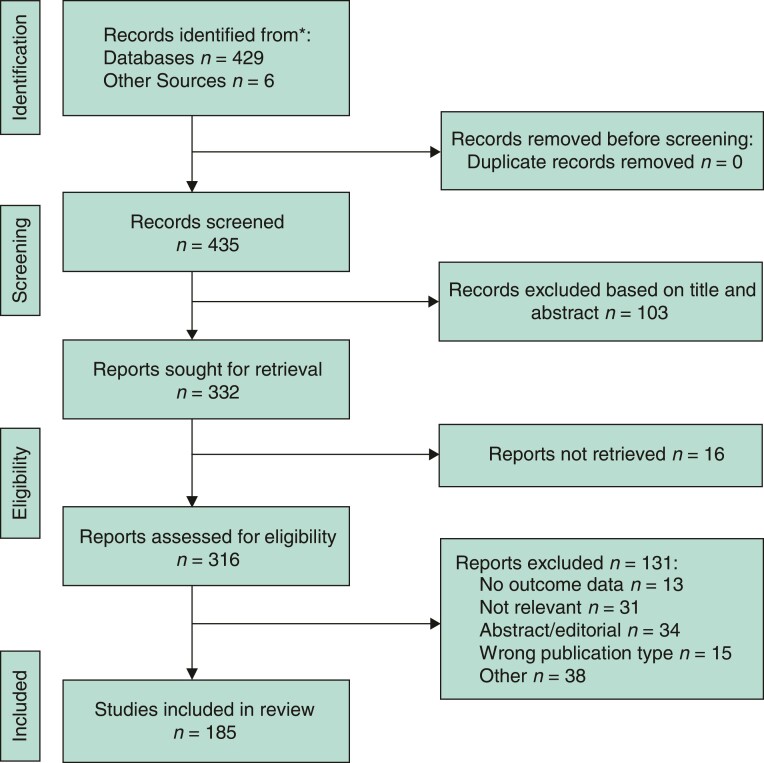
PRISMA flow diagram

The search of PubMed and Embase databases identified 429 unique articles, with a further six identified from a review of reference listings. A total of 103 articles were excluded on review of titles and abstracts. Sixteen reports could not be retrieved and a further 131 records were removed after full-text review, with 185 remaining for inclusion in the review (*Fig. 1*).

### Characteristics of included studies

The 185 included studies comprised 51 human studies (38 randomized clinical trials and 13 observational studies) and 134 animal studies. The type of surgery or animal respectively, and relative success of the barrier material are described in *Appendix S5*. Some 96 animal studies were in rat or mouse, 32 in rabbit, four in chicken, and two in pig. Human studies consisted of 26 gynaecological and 25 abdominal surgeries. Full details are described in *Appendix S6*.

### Characteristics of barrier agents

A total of 67 unique adhesion barriers materials were identified, comprising 16 barrier categories. The barrier materials included 37 natural and 30 synthetic products. The characteristics of the 67 barrier agents based on the eight distinctive properties are summarized in *Table 1* and described in detail in *Appendix S7*.

**Table 1:** Characteristics of promising barrier materials

	Adherence to traumatized tissue	Adherence to oozing tissue	Safety	Laparoscopic applicability	Ease of application	Postoperative pain	Cost-effectiveness
**ORC**	✓	✗	✓	✓	✓	✓	✓
**CMC/HA**	✓	✓	✓	✓	✓	✓	✓
**cHA**	✗ (Liquid)	✗ (Liquid)	✓	✓	✓	✓	✓
**Icodextrin**	✗ (Liquid)	✗ (Liquid)	✓	✓	✓	✓	✓
**PEG**	✗ (Liquid)	✗ (Liquid)	✓	✓	✓	✓	✓
**HA hydrogel**	✗ (Liquid)	✗ (Liquid)	✓	✓	✓	✓	?
**PLA/PEG**	✓	✗	?	✓	Mixed	Mixed	?
**Poloxamer 407/alginate**	✓	?	✓	✓	✓	?	?
**Dextran 70**	✓	✓	?	✓	?	?	?
**Polyester/collagen**	✓	✓	✓	✗	?	?	?

?, no data available. ORC, oxidized regenerated cellulose; CMC, carboxymethylcellulose; HA, hyaluronic acid; cHA, crosslinked HA; PEG, polyethylene glycol; PLA, polylactic acid.

#### Natural barriers

##### Algae

Alginate and alginate/hyaluronic acid both had success in animal studies^19–22^. No human studies were found for any of the materials. The alginate barrier had a higher efficacy compared with a commercialized barrier Interceed in an animal study^19^. Safety concerns for agar films were identified in an animal study, where there was an increased rate of adverse events^23^.

##### Cellulose

Oxidized regenerated cellulose (ORC) and a combination of carboxymethylcellulose (CMC)/hyaluronic acid (HA) had successful animal^24–48^ and human studies (*de novo*, reformation, elective, and emergency surgery) after both open and laparoscopic approaches^8,49–77^. ORC showed greater efficacy compared with control in reducing *de novo* adhesions during laparoscopic myomectomy^52^ but was inferior to poloxamer 407 in a comparator study^26^, although poloxamer 407 is only compatible on a completely haemolysed surface. ORC, modified xyloglucan hydrogel, and CMC/HA have very good safety profiles, low levels of postoperative pain, and score highly on ease of application^64,73,78^.

##### Chitosan

Six barriers identified had successful animal studies^79–88^ but had no human studies performed thus the safety profiles, cost-effectiveness, and levels of postoperative pain remain unknown.

##### Glycoprotein

Four barrier materials were identified as having successful findings in animal studies^36,39,40,89–95^, with only a single human study for fibrin, which was not successful in preventing *de novo* adhesions after open surgery^96^.

##### Hyaluronic acid

Three barriers were identified which were successful in animal studies^97–109^, with HA hydrogel achieving positive results in preventing *de novo* adhesions following laparoscopic surgery in a single human study^110,111^. It can be applied laparoscopically with low levels of postoperative pain^111^, although cost-effectiveness remains unknown.

##### Icodextrin

Icodextrin had positive outcomes in both animal^29,101,112^ and human studies (*de novo* and elective surgery)^113–117^. It can be applied laparoscopically and has positive outputs in terms of safety, cost-effectiveness, levels of postoperative pain, and ease of use^114,115,117^.

##### Starch

Sterile hydrophilic starch and dextrin had positive results in animal studies^29,118–120^, but neither material was successful in human studies^121,122^. Positive outputs have been reported for sterile hydrophilic starch in terms of safety, levels of postoperative pain, and ease of application^118–121^.

##### Miscellaneous

Twelve barriers in the group were identified with successful animal studies^102,123–136^; however, only Dextran 70 progressed to have a single successful human study (*de novo* and laparoscopic surgery). Each of the 12 barriers reported were easy to apply^102,117,124,126,132,136^; however, safety, cost-effectiveness, and levels of postoperative pain remain unknown for each barrier.

#### Synthetic barrier

##### Polycaprolactone

Four barriers had successful animal studies^137–144^, with no human studies identified. Polycaprolactone/polyhydroxybutyrate, and polycaprolactone/polyethylene glycol (PEG) can be applied laparoscopically and demonstrated good usability^141,145^.

##### Polyethylene glycol

Four barriers had successful animal studies^26,38,146–155^, with positive outcomes reported in human studies for PEG (*de novo*, reformation, and elective surgery) and poloxamer 407/alginate (*de novo*) in laparoscopic surgery^156–165^. No human studies were identified for poloxamer 407. PEG has had positive outputs in terms of patient safety, cost-effectiveness, and level of postoperative pain^157,159,160^. Poloxamer 407 alginate has been shown to have a high level of patient safety^165^, but cost-effectiveness, and postoperative pain are unknown.

##### Polyglycolic acid

The polyglycolic acid barrier had no successful animal study^166^ and no human studies have been identified.

##### Polylactic acid

Two barriers identified had successful animal studies^38,167–172^, with one successful human study performed analysing polylactic acid (PLA)/PEG barrier^173^. PLA/PEG had reports of high level of patient safety, mixed reports related to postoperative pain, and ease of application^169–171,173^.

##### Polypropylene

Polypropylene/omega-3 had a single successful animal study^174^, whereas the remaining three barriers in the category had unsuccessful animal studies^118,174^. No human studies were identified for any of the materials. Each of the barriers requires sutures to adhere to traumatized and oozing surfaces.

##### Polyvinyl alcohol

Polyvinyl alcohol hydrogel and polyvinyl alcohol/CMC had successful animal studies^175–181^, but no human studies were identified. Characteristics including patient safety, cost-effectiveness, and postoperative pain are unknown for the two barriers.

##### Silicone

Polysiloxane had no successful animal studies^182^ and no human studies have been performed to date^118,174,183^.

##### Miscellaneous

Eight further identified barriers except for polyester/collagen had successful animal studies^118,136,174,184–190^. No human studies were identified for any of the materials. Polyester/collagen has a poor level of safety reported in animal studies^118,174^, with unknown level of the ease of barrier application. Patient safety and ease of application are unknown for the remaining barriers.

### Pathway to market

The market potential for each barrier is described in *Table 2*, based on outcomes from animal and human studies. Six barriers with successful animal and human studies, which are currently available on the market were identified. A further 52 barrier materials with positive outcomes, where further research is required (success in both animal and human studies or success in animal studies without progression to human study) were identified. Fourteen barrier materials with negative outcomes were noted.

**Table 2:** Pathway to the market characteristics

Barrier type	Pathway status	Successful animal test	Followed by human test	Positive outputs	On the market
**Category**					
**Barrier name**					
**Natural**					
**Algae**					
Alginate	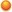	Yes	No	No	No
Agar films	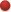	No	No	No	No
Alginate/hyaluronic acid	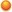	Yes	No	No	No
**Cellulose**					
Oxidized regenerated cellulose	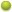	Yes	Yes	Yes	Yes
Modified xyloglucan hydrogel	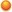	Yes	No	No	No
Carboxymethylcellulose	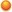	Yes	No	No	No
Carboxymethylcellulose/hyaluronic acid	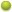	Yes	Yes	Yes	Yes
Carboxymethylcellulose/polyethylene glycol	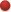	No	No	No	No
**Chitosan**					
N,O-carboxymethyl chitosan	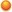	Yes	No	No	No
Chitosan	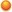	Yes	No	No	No
Chitosan/carboxymethylcellulose/collagen	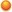	Yes	No	No	No
N,O-carboxymethyl chitosan/hyaluronic acid	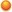	Yes	No	No	No
Chitosan/gelatin	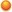	Yes	No	No	No
N,O-carboxymethyl chitosan/dextran	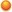	Yes	No	No	No
Chitosan/polyglycolic acid	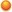	Yes	No	No	No
**Glycoprotein**					
Fibronectin derivative	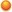	Yes	No	No	No
Lactoferrin	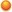	Yes	No	No	No
Fibrin	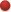	Yes	No	No	No
Gelatin/polyglycan	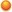	Yes	No	No	No
Gelatin/proteoglycan	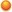	Yes	Yes	Yes	No
**Hyaluronic acid**					
Hyaluronic acid hydrogel	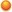	Yes	Yes	Yes	No
Crosslinked hyaluronic acid	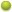	Yes	Yes	Yes	Yes
Hyaluronic acid membrane	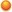	Yes	Yes	Yes	No
**Icodextrin**					
Icodextrin	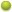	Yes	Yes	Yes	Yes
**Miscellaneous**					
Dextran 70	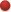	Yes	No	No	No
Phosphorylcholine	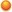	Yes	No	No	No
Silk	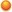	Yes	No	No	No
Ancrod	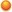	Yes	No	No	No
Bromelain	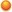	Yes	No	No	No
Xanthan gum	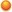	Yes	No	No	No
Pectin	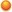	Yes	No	No	No
Modified pullulan	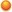	Yes	No	No	No
Liquid paraffin	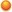	Yes	No	No	No
Galls ethyl acetate	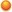	Yes	No	No	No
Ethyl pyruvate	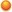	Yes	No	No	No
Tongfu xiere enteroclysis mixture	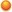	Yes	No	No	No
**Starch**					
Sterile hydrophilic starch	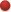	Yes	No	No	No
Dextrin	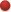	Yes	No	No	No
**Synthetic Polycaprolactone**					
Polycaprolactone/polyhydroxybutyrate	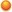	Yes	No	No	No
polycaprolactone/hyaluronic acid	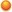	Yes	No	No	No
Polycaprolactone/polyethylene glycol	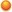	Yes	No	No	No
Polycaprolactone/gelatin	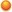	Yes	No	No	No
**Polyethylene glycol**					
Polyethylene glycol	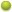	Yes	Yes	Yes	Yes
Polyethylene glycol/collagen/glycerol	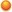	Yes	Yes	Yes	No
Poloxamer 407	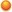	Yes	No	No	No
Poloxamer 407/alginate	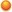	Yes	Yes	No	No
**Polyglycolic acid**					
Polyglycolic acid	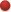	Yes	No	No	No
**Polylactic acid**					
Polylactic acid	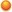	Yes	No	No	No
Polylactic acid/polyethylene glycol	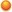	Yes	Yes	No	No
Polylactic acid/polycaprolactone	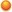	Yes	No	No	No
Poly(l-lactic acid)/modified mesoporous silica/ibuprofen	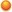	Yes	No	No	No
**Polypropylene**					
Polypropylene	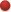	No	No	No	No
Polypropylene/glycolide/polycaprolactone	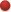	Yes	No	No	Yes
Polydioxanone/polypropylene/carboxymethylcellulose	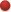	Yes	No	No	No
Polypropylene/titanium	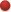	No	No	No	No
Polypropylene/omega 3	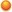	Yes	No	No	No
**Polyvinyl alcohol**					
Polyvinyl alcohol hydrogel	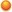	Yes	No	No	No
Polyvinyl Alcohol/carboxymethylcellulose	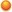	Yes	No	No	No
**Silicone**					
Polysiloxane		No	No	No	No
Polyesterurethane/polydimethylsiloxane	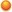	Yes	Yes	Yes	No
**Miscellaneous**					
Chitosan/poly(d,l-lactic-co-glycolic acid)/polyethylene oxide	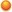	Yes	No	No	No
Polyester/collagen	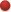	No	No	No	No
*N*-isopropylacrylamide	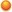	Yes	No	No	No
C17 glycerin ester	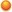	Yes	No	No	No
Methylene blue	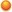	Yes	No	No	No
Dimethyl-sulfoxide	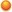	Yes	No	No	No
Polyhydroxyethylmethacrylate	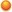	Yes	No	No	No
Poly(lactic-co-glycolic acid)/epigallocatechin-3-O-gallate	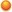	Yes	No	No	No

Green, on the market; orange, positive outcomes in animal and human study (but not on the market) or successful animal study with no human study to date; red, negative results from animal and/or human studies.

## Discussion

Ten barriers were identified (HA hydrogel, PLA/PEG, poloxamer 407/alginate, and Dextran 70 in addition to the six commercially available barriers) that achieved the primary outcome of preventing adhesions in both animal and human studies, with varying success in attaining each of the optimal characteristics. Furthermore, 48 additional barriers achieved positive outcomes in animal studies but never successfully progressed to a human study. The remaining nine barriers were those with unsuccessful human studies following positive animal studies and those with no successful in animal studies.

Animal models have been the basis of many great discoveries in modern biomedical research^14^; however, animal welfare must remain a central consideration. The large number of barriers achieving positive outcomes in animal subjects yet failing to progress to human trials questions the investigators’ intentions on progression, appropriateness of model utilized, study design, and reliability of results. Currently, there are six barriers available commercially in Europe comprising ORC (Interceed, Ethicon, Somerville, New Jersey, USA), CMC/HA (Seprafilm, Sanofi, Paris, France), crosslinked HA (cHA) (Hyalobarrier, Nordic group, Paris, France), polyester/collagen (Parietex, Medtronic, Watford, UK), icodextrin 4 per cent solution (Adept, Baxter, Deerfield, Illinois, USA), and PEG (Sprayshield, Integra, LifeSciences, Plainsboro, New Jersey, USA).

The capacity to adhere to traumatized tissue is a fundamental requirement for any barrier to envelope the damaged tissue and partition the aggregated fibrin surface, thereby diminishing adhesion formation^4^. Overall, only three natural (ORC, CMC/HA, and HA) and two synthetic (PLA/PEG and poloxamer 407/alginate) barriers that were successful in adhesion reduction in animal and human studies demonstrated adequate ability to adhere to traumatized tissue. The barrier was a liquid preparation, except for the PLA/PEG barrier, which requires sutures to impede migration. The PLA/PEG barrier has only been utilized in a single human study of cardiac patients with positive outcomes^173^; however, previous studies have shown that the additional use of sutures entails a heightened opportunity for adhesion formation^173,191^.

Barrier attachment to oozing surfaces is an important factor to ensure the anti-adhesion effect is maintained, particularly during surgeries that include a high risk of bleeding^148^. Overall, natural barriers seem to maintain more effective anti-adhesion effects on oozing surfaces. HA hydrogel and CMC/HA both highlighted their capabilities in human studies; however, the ORC barrier is of limited effectiveness in the presence of blood or peritoneal fluid^192^. Interestingly, chitosan-based (CS) barriers exhibit haemostatic effects^193^. This prophylactic property, in addition to the ability of the agent to be applied to oozing surfaces, highlights promise as a barrier constituent; however, although positive outputs were achieved in animal studies utilizing CS in combination^79–88,194,195^, no successful human study exists.

Patient safety is of utmost importance, balancing the utility risks of a barrier with the current standard of care (no barrier). Patients who suffer postoperative adhesions have a longstanding augmented risk of a number of discrete clinical sequelae, including chronic pain, small bowel adhesive disease, increased operating time, increased duration of hospital stay, female infertility, opioid dependency, and reduced quality of life^9,196^. While, any potential barrier candidate should aim to alleviate or reduce potential patient risks, it is important that the barrier itself does not pose further patient safety concerns or augment postoperative pain. Overall, the nine barriers achieving the primary endpoint of reducing the extent and severity of postoperative adhesions scored highly on the Likert safety scale. Five barriers achieved positive results regarding extent of postoperative pain, with PLA/PEG barrier having mixed results, whereas poloxamer/alginate and Dextran 70 barriers had no reported outcomes.

The application of ORC during gynaecological surgery decreases the incidence and severity of postoperative adhesions without any significant adverse events^76^. Concerns have been raised that a single adhesion band produced from incomplete cover or on the periphery of a barrier may result in an augmented risk of strangulated SBO; however, the available evidence contradicts these concerns, highlighting that extensive adhesive disease as opposed to isolated areas correlates with incidence of SBO^7^. The CMC/HA barrier has been demonstrated to reduce the rate of SBO in several controlled trials^56,59^. Furthermore studies have found a reduction in the incidence of chronic abdominal pain^8^ and duration of procedure^77^. Despite predominantly positive outputs for the barrier, safety concerns have been highlighted with augmented risk of abdominal abscess formation on application of the barrier to the region of anastomoses^56,62^.

The utilization of a laparoscopic approach, where feasible, has consistently demonstrated improved patient outcomes relative to open surgery. Krielen and colleagues analysed a retrospective cohort study of 72 270 patients with adhesion-related readmissions following abdominal surgery, comprising open (*n* = 50 751) and laparoscopic (*n* = 21 519) approaches. The study interval encompassed hospital readmissions from 2009 to 2011 utilizing the validated population data for the Scottish National Health Service with a 5-year follow-up. They recorded a statistically significant reduction in the number of readmissions directly related to adhesions (1.7 per cent *versus* 4.3 per cent; *P* < 0.0001) and those possibly related to adhesions (16.0 per cent *versus* 18.2 per cent; *P* < 0.005) in the laparoscopic group^6^. Of the nine barriers highlighted, each can be applied laparoscopically except for PLA/PEG, where it is unknown and mixed results are reported regarding its ease of application. No studies to date have reported the ease of application of Dextran 70. ORC and CMC/HA are solid membrane barriers and therefore present an augmented challenge in laparoscopic application compared with alternative barriers, which are liquid, gel, or spray preparations. ORC has also been associated with elevated handling issues in comparison with the other preparations.

Postoperative adhesions and related complications accrue substantial healthcare costs, both directly and indirectly. Cost-effectiveness analysis of widespread utilization is an essential prerequisite for any barrier considered for introduction by policymakers. No such analysis assessing the overall cost-effectiveness of a barrier was identified in this systematic review.

The primary strength of the present study is that independent screening and abstraction for both animal and human studies was performed, resulting in the largest systematic review on the topic to date. Ideal characteristics for each barrier were independently reviewed and extracted, allowing potential barriers to be highlighted for further investigation; however, limitations including publication bias and small study bias exist as with all systematic reviews. Additional limitations rely on heterogenous reporting of characteristics and study success. Furthermore, animal models and human clinical indications were heterogenous. It was not possible to assess the long-term safety and efficacy data of the majority of barriers, as most only included short-term data.

Meticulous surgical technique and increasing performance of minimally invasive procedures have reduced the incidence and severity of the complication, but adhesions remain a significant global burden. Despite a concerted effort and vast investigation over the past two decades, there remains no specific barrier agent in widespread use internationally with only five agents licenced for use in the EU. Positive long-term data on efficacy and safety have been demonstrated for Seprafilm^8^; however, these remain sparse overall. Future research should concentrate on assessing the safety and confirming efficacy observed in animal studies, ensuring that all research is well designed, relevant, and takes into account issues on animal welfare. Outcomes should be reported in a uniform manner based on location of adhesions (such as the modified American Fertility Society endometriosis scale for gynaecology adhesions). Effects on quality of life seem to have been poorly explored to date and require evaluation. Furthermore, before the production of novel barriers, researchers must first ensure compliance with the EU Directive guidance, which puts a clear and explicit obligation on researchers to replace, reduce, and refine studies with animal involvement^15^. Additionally, alignment with clinically based surgeons to identify and assess reluctance and possible concerns with utilization of commercially available barriers, including Seprafilm, is required, and the long-term efficiency and safety data of successful barriers requires evaluation in future research^8^.

## Funding

The authors have no funding to declare.

## Supplementary Material

zrac075_Supplementary_Data

## Data Availability

The data that support the findings of this study are available from the corresponding author, M.W., upon reasonable request.
