# Supplementary material for: Barrier materials for prevention of surgical adhesions: systematic review
Source: BJS Open. 2022 Jun 6;6(3):zrac075. doi: 10.1093/bjsopen/zrac075 (PMC9167938; doi:10.1093/bjsopen/zrac075)
Supplement: zrac075_Supplementary_Data [file zrac075_supplementary_data.zip › Supplementary_material.docx]

## ***Supplementary material***

## Appendix S1 – Study Protocol

| TITLE | CHECKLIST ITEM | REPORTED ON PAGE # |
| --- | --- | --- |
| 1. Title: | Title Systemic Review of materials for prevention of surgical adhesions  Registration PROSPERO <https://www.crd.york.ac.uk/prospero/> |  |
| **BACKGROUND** |  |  |
| 2. Objectives: | The research question including components such as participants, interventions, comparators, and outcomes.  The formation of adhesions is a frequent complication of abdominal surgery, which occurs in a reported 90% of patients post laparotomy (1), with re-admissions directly related to adhesions reported as 5% over five years (2). Although the majority of patients with adhesions remain asymptomatic, a substantial number develop symptomatic “adhesive disease” with significant ensuing morbidity, which is characteristically difficult to treat (3) and entails a significant financial burden on the health system (4). Thus prevention or reduction of their formation is a key priority. Many materials have been evaluated as physical barriers to separate wounded from surrounding tissue to reduce the rate post-operative adhesions, the most frequently studied been ‘membrane barriers’ consisting of a variety of materials (5). Despite this there is no published information characterising and comparing the different materials.  **Objective**  To conduct a systematic review of the materials used for prevention of surgical adhesions.  **Specific Objectives**   1. Characterisation of materials in the published literature for prevention of surgical adhesions 2. Comparison to the ideal solution (criteria to be defined) 3. Recommendation for further work  - Materials to progress for further research - Potential combination of pre-existing materials |  |
| **METHODS** |  |  |
| 3. Eligibility criteria: | Study and report characteristics used as criteria for inclusion.  **Types of Studies**  Randomised controlled trials.  Observational studies.  Any measure of outcome.  **Types of participants**  Human or animal.  Unrestricted age, gender, condition, surgery type.  **Inclusion Criteria**  Published studies (MEDLINE, EMBASE, CENTRAL)  Published any time before February 2019.  English Language. |  |
| 4. Information sources: | Key databases searched and search dates.  **Electronic Searches**  MEDLINE (PUBMED)  EMBASE  Cochrane Central Register of Controlled Trials (CENTRAL)  **Search Date**  04/02/2019  **Other Searches**  In addition, hand searches will be conducted of the reference lists of all articles retrieved to identify  other potentially eligible articles.  **Search Strategy**  Pubmed/MEDLINE  Surg*[Title/Abstract] AND adhesion*[Title/Abstract] AND prevention*[Title/Abstract]  MeSH Terms (MEDLINE)  EMBASE (EMTREE)  **Strategies to Minimize Bias**  Cochrane Highly Sensitive Search Strategies for identifying randomized trials and non-randomized clinical studies in MEDLINE  **Data extraction**  Three Reviewers to review independently   1. Michael Waldron 2. Conor Judge 3. Laura Farina   **Extraction Variables**   1. Year of publication 2. Journal 3. Study design 4. Quality of study (Jadad and MINORS score) 5. Surgical Speciality 6. Funder 7. Condition 8. Outcome 9. Intervention 10. Number of participants 11. Country of trial 12. Characteristics of Material 13. Chemical composition |  |
| 5. Risk of bias: | Methods of assessing risk of bias.  As per Cochrane Risk of Bias Tool  Method of random sequence generation  Concealment of treatment  Blinding (participants/care providers/assessors)  Use of intention to treat analysis. |  |
| **RESULTS** |  |  |
| 6. Included studies: | Number and type of included studies and participants and relevant characteristics of studies. |  |
| 7. Synthesis of results: |  |  |
| 8. Description of the effect: |  |  |
| **DISCUSSION** |  |  |
| 9. Strengths and Limitations of evidence: | Brief summary of strengths and limitations of evidence (e.g. inconsistency, imprecision, indirectness, or risk of bias, other supporting or conflicting evidence) |  |
| 10. Interpretation: | General interpretation of the results and important implications |  |
| **OTHER** |  |  |
| 11. Funding: | Primary source of funding for the review. |  |
| 12. Registration: | Registration number and registry name. |  |

## Appendix S2 – Data Collection Forms

1. **Barrier Specific**

| Barrier Number | Barrier Type (Natural/Synthetic) | Barrier Category | Combination (Y/N) | Barrier Name | Brand Name | Mechanism of Action | Composition | Solid-liquid-gel |
| --- | --- | --- | --- | --- | --- | --- | --- | --- |
|  |  |  |  |  |  |  |  |  |

1. **Animal Study Specific**

| Barrier Number | Animal Title | Animal DOI | Animal Year | Animal Species | Successful Study | Comment |
| --- | --- | --- | --- | --- | --- | --- |
|  |  |  |  |  |  |  |

| Barrier Number | Human Title | Human DOI | Human Year | Human Study Type  (Observation study/randomised clinical study) |
| --- | --- | --- | --- | --- |
|  |  |  |  |  |

1. **Human Study Specific**

Legend: 1. Form of information collected for each barrier agent. 2. Form of information collected from each animal study. 3. Form of information collected from each human study

## Appendix S3 – Desirable Barrier Characteristics Interpretation

| **Desirable Barrier characteristic** | **Measurement** | **Interpretation** |
| --- | --- | --- |
| Adherence to traumatized tissue | Yes/no, comment reason if ‘no’ (if known) | Yes = Adheres  No = Fails to adhere |
| Adherence to oozing surface | Yes/no, comment reason if ‘no’ (if known) | Yes = Adheres  No = Fails to adhere |
| Application though laparoscope | Yes/no/unknown | Yes = Possible to apply laparoscopically  No = Not possible to apply laparoscopically  Unknown =Application laparoscopically uncertain |
| Safety for patient | Likert scale (1-5/unknown) | Likert scale = rating from one to five for each barrier, dependent on harm to patient reported in human study   - 1 = death/severe complication(s), - 2 = moderate to severe complication(s), - 3 = moderate complication(s), - 4 = mild/moderate complication(s), - 5 = no/mild complication(s)). - If no human study performed from which safety could be assessed, then safety was recorded as unknown |
| Cost effectiveness | Likert scale (1-5/unknown) | Likert scale = rating from one to five for each barrier, dependent on estimated cost effectiveness   - 1 = Very poor, - 2 = poor, - 3 = neutral, - 4 = high, - 5 = very high - If no human study with documented cost assessment performed, then cost effectiveness was recorded as unknown |
| Post-operative pain | Likert scale (1-5/unknown): | Likert scale = rating from one to five for each barrier, dependent on reported pain by subjects in studies of human subjects   - 1 = Very poor, - 2 = poor, - 3 = neutral, - 4 = high, - 5 = very high - If no human study with documented measurement of post-operative pain performed, then post-operative pain was recorded as unknown. |
| Ease of application | Likert scale (1-5/unkown) | Likert scale = rating from one to five dependent how difficult the barrier is to apply   - 1 = Very poor, - 2 = poor, - 3 = neutral, - 4 = high, - 5 = very high - If no human study whereby difficulty in application could be assessed, then ease of application was recorded as unknown. |

## Appendix S4 – Pathway to Market Characteristics Interpretation

| **Pathway to the Market characteristic** | **Reported** | **Interpretation** |
| --- | --- | --- |
| Successful animal study | Yes/No | Yes = one or more animal study with positive outputs.  No = Zero animal study with positive outputs. |
| Human study performed | Yes/No | Yes = one or more human studies performed.  No = Zero human studies performed. |
| Positive finding in human study | Yes/No | Yes = one or more human study with positive outputs.  No = Zero human study with positive outputs. |
| Product on the market | Yes/No | Yes = Product is currently available commercially.  No = Product is not currently available commercially. |

## Appendix S5 – Study Type, participants and relative success of the barrier agent

|  |  |  | **Animal Studies** | |  |  | **Human Studies** | |  |  |  |
| --- | --- | --- | --- | --- | --- | --- | --- | --- | --- | --- | --- |
| **Barrier Type** | | | **Study Count** | **Successful Study Count** |  |  | **Study Count** | **Abdominal Count** | **Gynae Count** | **Observational Count** | **RCT Count** |
|  | **Category** | |  |  |  |  |  |  |  |  |  |
|  |  | **Barrier Name** |  |  |  |  |  |  |  |  |  |
| **Natural** | | |  |  |  |  |  |  |  |  |  |
|  | **Algae** | |  |  |  |  |  |  |  |  |  |
|  |  | Alginate | 4 | 4 |  |  |  |  |  |  |  |
|  |  | Agar films | 1 | 0 |  |  |  |  |  |  |  |
|  |  | Alginate/ Hyaluronic Acid | 1 | 1 |  |  |  |  |  |  |  |
|  | **Cellulose** | |  |  |  |  |  |  |  |  |  |
|  |  | Single |  |  |  |  |  |  |  |  |  |
|  |  | Oxidised regenerated cellulose | 11 | 6 |  |  | 6 | 1 | 5 | 0 | 6 |
|  |  | Modified xyloglucan hydrogel | 2 | 2 |  |  |  |  |  |  |  |
|  |  | Carboxymethylcellulose | 4 | 4 |  |  |  |  |  |  |  |
|  |  | Combination |  |  |  |  |  |  |  |  |  |
|  |  | Carboxymethylcellulose / Hyaluronic Acid | 20 | 20 |  |  | 21 | 12 | 9 | 7 | 14 |
|  |  | Carboxymethylcellulose / Polyethylene Glycol | 1 | 0 |  |  |  |  |  |  |  |
|  | **Chitosan** | |  |  |  |  |  |  |  |  |  |
|  |  | Single |  |  |  |  |  |  |  |  |  |
|  |  | N,O-carboxymethyl chitosan | 1 | 1 |  |  |  |  |  |  |  |
|  |  | Hydroxybutyl Chitosan | 2 | 2 |  |  |  |  |  |  |  |
|  |  | Combination |  |  |  |  |  |  |  |  |  |
|  |  | Chitosan/ Carboxymethylcellulose /collagen | 2 | 2 |  |  |  |  |  |  |  |
|  |  | N,O-carboxymethyl chitosan/ Hyaluronic Acid | 2 | 2 |  |  |  |  |  |  |  |
|  |  | Chitosan / gelatin | 2 | 1 |  |  |  |  |  |  |  |
|  |  | N,O-carboxymethyl chitosan/Dextran | 1 | 1 |  |  |  |  |  |  |  |
|  | **Glycoprotein** | |  |  |  |  |  |  |  |  |  |
|  |  | Single |  |  |  |  |  |  |  |  |  |
|  |  | Lactoferrin | 1 | 1 |  |  |  |  |  |  |  |
|  |  | Fibrin | 4 | 3 |  |  | 1 | 0 | 1 | 1 | 0 |
|  |  | Combination |  |  |  |  |  |  |  |  |  |
|  |  | Gelatin derivative | 2 | 2 |  |  |  |  |  |  |  |
|  |  | Gelatin/proteoglycan | 2 | 2 |  |  |  |  |  |  |  |
|  | **Hyaluronic Acid** | |  |  |  |  |  |  |  |  |  |
|  |  | Single |  |  |  |  |  |  |  |  |  |
|  |  | Hyaluronic acid Hydrogel | 5 | 4 |  |  | 2 | 0 | 1 | 0 | 2 |
|  |  | Crosslinked Hyaluronic Acid | 4 | 4 |  |  | 2 | 0 | 2 | 1 | 1 |
|  |  | Hyaluronic Acid membrane | 1 | 1 |  |  |  |  |  |  |  |
|  | **Icodextrin** | |  |  |  |  |  |  |  |  |  |
|  |  | Single |  |  |  |  |  |  |  |  |  |
|  |  | Icodextrin | 3 | 2 |  |  | 5 | 2 | 3 | 0 | 5 |
|  | **Miscellaneous** | |  |  |  |  |  |  |  |  |  |
|  |  | Single |  |  |  |  |  |  |  |  |  |
|  |  | Dextran 70 | 1 | 1 |  |  | 1 | 0 | 1 | 0 | 1 |
|  |  | Phosphorylcholine | 3 | 3 |  |  |  |  |  |  |  |
|  |  | Silk | 1 | 1 |  |  |  |  |  |  |  |
|  |  | Ancrod | 1 | 1 |  |  |  |  |  |  |  |
|  |  | Bromelain | 1 | 1 |  |  |  |  |  |  |  |
|  |  | Xanthan gum | 1 | 1 |  |  |  |  |  |  |  |
|  |  | Pectin | 3 | 3 |  |  |  |  |  |  |  |
|  |  | Modified pullulan | 1 | 1 |  |  |  |  |  |  |  |
|  |  | Liquid paraffin | 1 | 1 |  |  |  |  |  |  |  |
|  |  | Galls ethyl acetate | 1 | 1 |  |  |  |  |  |  |  |
|  |  | Ethyl pyruvate | 1 | 1 |  |  |  |  |  |  |  |
|  |  | Tongfu Xiere Enteroclysis Mixture | 1 | 1 |  |  |  |  |  |  |  |
|  | **Starch** | |  |  |  |  |  |  |  |  |  |
|  |  | Single |  |  |  |  |  |  |  |  |  |
|  |  | Sterile hydrophilic starch | 3 | 3 |  |  | 1 | 1 | 0 | 1 | 0 |
|  |  | Dextrin | 1 | 1 |  |  | 1 | 1 | 0 | 1 | 0 |
| **Synthetic** | | |  |  |  |  |  |  |  |  |  |
|  | **Miscellaneous** | |  |  |  |  |  |  |  |  |  |
|  |  | Chitosan/ Poly(d,l-lactic-co-glycolic acid)/ Polyethylene oxide | 1 | 1 |  |  |  |  |  |  |  |
|  |  | Polyester/collagen | 2 | 0 |  |  |  |  |  |  |  |
|  |  | C17 glycerin ester | 1 | 1 |  |  |  |  |  |  |  |
|  |  | Methyline blue | 1 | 1 |  |  |  |  |  |  |  |
|  |  | Dimethyl-sulfoxide | 1 | 1 |  |  |  |  |  |  |  |
|  |  | Poly(lactic-co-glycolic acid)/ Epigallocatechin-3-O-gallate | 1 | 1 |  |  |  |  |  |  |  |
|  | **Polycaprolactone** | |  |  |  |  |  |  |  |  |  |
|  |  | Combination |  |  |  |  |  |  |  |  |  |
|  |  | Polycaprolactone/Polyhydroxybutyrate | 1 | 1 |  |  |  |  |  |  |  |
|  |  | Polycaprolactone/Hyaluronic Acid | 2 | 2 |  |  |  |  |  |  |  |
|  |  | Polycaprolactone/Polyethylene Glycol | 4 | 4 |  |  |  |  |  |  |  |
|  |  | Polycaprolactone/Gelatin | 1 | 1 |  |  |  |  |  |  |  |
|  | **Polyethylene Glycol** | |  |  |  |  |  |  |  |  |  |
|  |  | Single |  |  |  |  |  |  |  |  |  |
|  |  | Polyethylene Glycol | 5 | 5 |  |  | 9 | 5 | 4 | 0 | 9 |
|  |  | Poloxamer 407 | 4 | 3 |  |  |  |  |  |  |  |
|  |  | Combination |  |  |  |  |  |  |  |  |  |
|  |  | Poloxamer 407/alginate | 2 | 2 |  |  | 1 | 1 | 0 | 0 | 1 |
|  | **Polyglycolic Acid** | |  |  |  |  |  |  |  |  |  |
|  |  | Single |  |  |  |  |  |  |  |  |  |
|  |  | Polyglycolic Acid | 1 | 0 |  | | |  |  |  |  |
|  | **Polylactic Acid** | |  |  |  |  |  |  |  |  |  |
|  |  | Single |  |  |  |  |  |  |  |  |  |
|  |  | Polylactic Acid | 3 | 2 |  |  |  |  |  |  |  |
|  |  | Combination |  |  |  |  |  |  |  |  |  |
|  |  | Polylactic Acid / Polyethylene Glycol | 3 | 3 |  |  |  |  |  |  |  |
|  | **Polypropylene** | |  |  |  |  |  |  |  |  |  |
|  |  | Single |  |  |  |  |  |  |  |  |  |
|  |  | Polypropylene | 1 | 0 |  |  |  |  |  |  |  |
|  |  | Combination |  |  |  |  |  |  |  |  |  |
|  |  | Polypropylene/glycolide/Polycaprolactone | 1 | 0 |  |  |  |  |  |  |  |
|  |  | Polydioxanone/polypropylene/Carboxymethylcellulose | 2 | 0 |  |  |  |  |  |  |  |
|  |  | Polypropylene/Titanium | 1 | 0 |  |  |  |  |  |  |  |
|  |  | Polypropylene/Omega 3 | 1 | 1 |  |  |  |  |  |  |  |
|  | **Polyvinyl Alcohol** | |  |  |  |  |  |  |  |  |  |
|  |  | Single |  |  |  |  |  |  |  |  |  |
|  |  | Polyvinyl Alcohol hydrogel | 4 | 4 |  |  |  |  |  |  |  |
|  |  | Combination |  |  |  |  |  |  |  |  |  |
|  |  | Polyvinyl Alcohol/ Carboxymethylcellulose | 2 | 2 |  |  |  |  |  |  |  |
|  | **Silicone** | |  |  |  |  |  |  |  |  |  |
|  |  | Single |  |  |  |  |  |  |  |  |  |
|  |  | Polysiloxane (61) | 1 | 0 |  |  |  |  |  |  |  |

## Appendix S6 – Characteristics of Included Barriers

### Natural Barrier

#### Material 1 – Algae

Three barriers were identified in the *Algae* group; agar film, alginate and alginate/ hyaluronic acid. Each of the barriers can adhere to traumatised tissue. Both alginate and alginate/ hyaluronic acid can adhere to oozing tissue and can be applied laparoscopically (17–19,238), whilst it is uncertain from current literature as to the characteristics of agar film. Safety concerns for agar films were identified in an animal study, where there was an increased rate of adverse events (21). Although no safety concerns were identified for the other barriers in the group. No human studies were performed for the three barriers, thereby no conclusions can be drawn regarding patient safety, cost-effectiveness, post-operative pain or ease of application.

#### Material 2 - Cellulose

Five barriers were identified in the *Cellulose* group; oxidised regenerated cellulose, modified xyloglucan hydrogel, carboxymethylcellulose, carboxymethylcellulose/ hyaluronic acid and carboxymethylcellulose/ polyethylene glycol. Oxidised regenerated cellulose, modified xyloglucan hydrogel, carboxymethylcellulose and carboxymethylcellulose/ hyaluronic acid barriers are capable of adhering to traumatised tissue (32,74,81,235), whilst carboxymethylcellulose/ polyethylene glycol barriers were not as it is a liquid preparation (175). The carboxymethylcellulose/ hyaluronic acid barrier can be applied to oozing surfaces (65), but it is unknown whether xyloglucan hydrogel is adherent to oozing surfaces, whilst the remaining three barriers are not capable. The five barriers can be applied laparoscopically (12,65,169). Oxidised regenerated cellulose, modified xyloglucan hydrogel and carboxymethylcellulose/ hyaluronic acid have very good safety profiles, low levels of post-operative pain and score highly on ease of application (67,76,81). The three barriers are available on the market, but cost effectiveness remains uncertain. The remaining two barrier carboxymethylcellulose and carboxymethylcellulose/ polyethylene glycol had no human studies identified, so the safety, cost-effectiveness, degree of post-operative pain and ease of application are unknown.

#### Material 3 - Chitosan

Eight barriers were identified in the *Chitosan* group; N,O-carboxymethyl chitosan, hydroxybutyl chitosan, chitosan/ carboxymethylcellulose/ collagen, N,O-carboxymethyl chitosan/ hyaluronic acid, chitosan/ gelatin, N,O-carboxymethyl chitosan/ dextran, chitosan/ poly(L-glutamic acid) and chitosan/ pectin. N,O-carboxymethyl chitosan, hydroxybutyl chitosan, chitosan/ carboxymethylcellulose/ collagen, N,O-carboxymethyl chitosan/ dextran, poly(L-glutamic acid) and chitosan/ pectin barrier are capable of adhering to traumatised tissue (83,84,86,92,93), whilst N,O-carboxymethyl chitosan/ hyaluronic acid and chitosan/ gelatin barriers are not as they are liquid preparations. N,O-carboxymethyl chitosan, hydroxybutyl chitosan, chitosan/ carboxymethylcellulose/ collagen and chitosan/ pectin can be applied to oozing surfaces (82,84,86,149), but it is unknown whether N,O-carboxymethyl chitosan/ hyaluronic acid, N,O-carboxymethyl chitosan/ dextran, poly(L-glutamic acid) are able, whilst chitosan/ dextran is not capable (92). N,O-carboxymethyl chitosan/ hyaluronic acid, chitosan/ gelatin and N,O-carboxymethyl chitosan/ dextran can be applied laparoscopically, whilst it is unknown regarding the remaining barriers. N,O-carboxymethyl chitosan/ hyaluronic acid and chitosan/ gelatin barriers offer very high ease of application being liquid preparations (88,90), whilst the remaining barriers ease of application is unknown. The safety profiles, cost-effectiveness and levels of post-operative pain remain unknown as no human studies were identified.

#### Material 4 – Glycoprotein

Five barriers were identified in the *Glycoprotein* group; fibronectin derivative, lactoferrin, gelatin/ proteoglycan, gelatin/ polyglycan ester and fibrin. Fibronectin, gelatin/ polyglycan ester and fibrin can adhere to traumatized tissue. Gelatin/ proteoglycan and fibrin can be applied to oozing tissue, while this remains unknown regarding fibronectin derivative (106,109,117). The remaining two barriers lactoferrin and gelatin/ proteoglycan are liquid preparations, which can’t adhere to traumatised or oozing tissue, but can both be applied laparoscopically and easily (107,109). It is unknown if they can be applied laparoscopically and the ease of use for the other three barriers. The safety, cost-effectiveness and post-operative characteristics can’t be determined.

#### Material 5 – Hyaluronic acid

Three barriers were identified in the *Hyaluronic acid* group; hyaluronic acid hydrogel, cross-linked hyaluronic acid and hyaluronic acid membrane. Hyaluronic acid hydrogel can be applied to traumatised and oozing tissue, while cross-linked hyaluronic acid can’t as it is a liquid preparation and hyaluronic acid membrane requires a suture to prevent migration. Hyaluronic acid hydrogel and cross-linked hyaluronic acid can be applied laparoscopically with low levels of post-operative pain, whilst hyaluronic acid membrane can’t be applied via laparoscopic procedure and level of post-operative pain remains unknown. Each of the barriers have shown to be safe and easy to apply (132,134,135,239). Cross-linked hyaluronic acid has been reported to be a cost-effective intervention (240), whilst the cost-effectiveness is uncertain for the other barriers.

#### Material 6 – Icodextrin

One barrier was identified in the *Icodextrin* group; Icodextrin. Icodextrin doesn’t adhere to traumatised or oozing surfaces as it is a liquid preparation. It can be applied laparoscopically and has positive outputs in terms of safety, cost-effectiveness, levels of post-operative pain and ease of use (140,141,143).

#### Material 7 – Miscellaneous

Twelve barriers were identified in the *Miscellaneous* group; Dextran 70, silk, ancrod, bromelain, xanthan gum, pectin, modified pullulan, liquid paraffin, galls ethyl acetate, ethyl pyruvate, Tongfu Xiere enteroclysis and phosphorylcholine. Dextran 70, ancrod, modified pullulan and galls ethyl acetate adhere to traumatized and oozing tissue, whilst xanthan gum, pectin can adhere to traumatised but unknown regarding adherence to oozing tissue (133,143,152,153,157,164). Silk, bromelain, liquid paraffin, ethyl pyruvate, Tongfu Xierre Enteroclysis and phosphorylcholine are liquid preparations, which can’t adhere to traumatised or oozing tissue. Each of the barriers can be applied laparoscopically, with the exception of silk which is unknown. The twelve barriers are reported to be easy to apply (126,143,151,153,159,164). However, safety, cost-effectiveness and levels of post-operative pain remain unknown for each barrier.

### Synthetic Barrier

#### Material 1 – Polycaprolactone (PCL)

Four barriers were identified in the *Polycaprolactone* group; polycaprolactone/ polyhydroxybutyrate, polycaprolactone/ hyaluronic acid, polycaprolactone/ polyethylene glycol and polycaprolactone/ gelatin. Polycaprolactone/ hyaluronic acid and polycaprolactone/ polyethylene glycol can adhere to traumatised tissue whilst polycaprolactone/ polyhydroxybutyrate and polycaprolactone/ gelatin require suturing to prevent migration of the barrier (166,168,171,174). Polycaprolactone/ polyethylene glycol can adhere to oozing tissue, but it is unknown whether polycaprolactone/ hyaluronic acid can do so. Polycaprolactone/ polyhydroxybutyrate and polycaprolactone/ polyethylene glycol can be applied laparoscopically and have a high level of ease of use (168,170), while this remains unknown for the additional two barriers. Characteristics related to barrier safety, cost-effectiveness and post-operative pain remain unknown for each of the barriers.

#### Material 2 – Polyglycolic Acid (PGA)

One barrier was identified in the *Polyglycolic Acid* group; Polyglycolic Acid. The barrier requires sutures to adhere to traumatised and oozing tissue (199). Characteristics including application laparoscopically, patient safety, cost-effectiveness and post-operative pain are unknown.

#### Material 3 – Polyethylene Glycol (PEG)

Three barriers were identified in the *Polyethylene Glycol* group; polyethylene glycol, poloxamer 407 and poloxamer 407/ alginate. Poloxamer 407 and poloxamer 407/ alginate adhere to traumatised tissue, but it is uncertain whether either can be applied to oozing surfaces, while polyethylene glycol is incapable as it is a liquid preparation. The three barriers can be applied laparoscopically and have a high level of ease of use. Polyethylene glycol has had positive outputs in terms of patient safety, cost-effectiveness and level of post-operative pain (190,192,193). Poloxamer 407/ alginate has been shown to have a high level of patient safety (198), but cost-effectiveness and post-operative pain are unknown. The three characteristics remain unknown for Poloxamer 407 with no human studies identified for the barrier.

#### Material 4 – Polylactic Acid (PLA)

Four barriers were identified in the *Polylactic Acid* group; polylactic acid, polylactic acid/ polyethylene glycol, polylactic acid/ polycaprolactone and poly(l-lactic acid)/ modified mesooporous silica/ ibuprofen. Polylactic acid/ polycaprolactone can adhere to traumatised and oozing surfaces, while poly(l-lactic acid)/ modified mesooporous silica/ ibuprofen can adhere to traumatised but unknown related to oozing surfaces (207,208). The remaining two barriers do not adhere, with polylactic acid requiring sutures to prevent migration (40). Polylactic acid, polylactic acid/ polycaprolactone and poly(l-lactic acid)/ modified mesooporous silica/ ibuprofen can’t be applied laparoscopically, while it is unknown for polylactic acid/ polyethylene glycol. Polylactic acid/ polyethylene glycol has had reports of high level of patient safety, mixed reports related to post-operative pain and ease of application (203–205,209). Characteristics including patient safety, cost-effectiveness and post-operative pain are unknown for the remaining three barriers.

#### Material 5 – Polypropylene

Five barriers were identified in the *Polypropylene* group; polypropylene, polypropylene/ glycolide/ polycaprolactone, polydioxanone/ polypropylene/ carboxymethylcellulose, polypropylene/ titanium and polypropylene/ omega 3. Each of the barriers requires sutures to adhere to traumatised and oozing surfaces. Polypropylene/ glycolide/ polycaprolactone and polydioxanone/ polypropylene/ carboxymethylcellulose can be applied laparoscopically (210), while it is unknown for the other three barriers. Polydioxanone/ polypropylene/ carboxymethylcellulose has had positive results in terms of patient safety, post-operative pain and ease of application. Polypropylene/ glycolide/ polycaprolactone has had mixed reports related to patient safety, with post-operative pain and ease of application remaining uncertain (210). Patient safety, post-operative pain and ease of application are unknown for the remaining three barriers. Cost-effectiveness is unknown for each of the barriers.

#### Material 6 – Polyvinyl Alcohol (PVA)

Two barriers were identified in the *Polyvinyl Alcohol* group; polyvinyl alcohol hydrogel and polyvinyl alcohol/ carboxymethylcellulose. Both of the barriers are capable of adhering to traumatised tissue (216,217). Polyvinyl alcohol/ carboxymethylcellulose can adhere to oozing tissue and be administered laparoscopically, whilst both are unknown for polyvinyl alcohol hydrogel. The two barriers have a high level of ease of application (216). Characteristics including patient safety, cost-effectiveness and post-operative pain are unknown for the two barriers.

#### Material 7 – Silicone

Two barriers were identified in the *Silicone* group; polysiloxane and polydimethylsiloxane/ polyesterurethane. Polysiloxane can be applied to traumatised tissue, whilst it is unknown whether it can be applied to oozing surfaces. Polydimethylsiloxane/ polyesterurethane requires sutures to adhere and prevent migration of the barrier (50,218). Characteristics including application laparoscopically, patient safety, cost-effectiveness and post-operative pain are unknown for the two barriers.

#### Material 8 – Miscellaneous

Eight barriers were identified in the *Miscellaneous* group; Chitosan/ poly(d,l-lactic-co-glycolic acid)/ polyethylene oxide, polyester/ collagen, N-isopropylacrylamide, C17 glycerin ester, methylene blue, dimethyl-sulfoxide, polyhydroxyethylmethacrylate and poly(lactic-co-glycolic acid)/ epigallocatechin-3-O-gallate. Chitosan/ poly(d,l-lactic-co-glycolic acid)/ polyethylene oxide, polyester/ collagen, N-isopropylacrylamide, C17 glycerin ester, methylene blue and polyhydroxyethylmethacrylate can adhere to traumatised tissue (144,221,223–225), whilst dimethyl-sulfoxide is a liquid preparation and poly(lactic-co-glycolic acid)/ epigallocatechin-3-O-gallate requires sutures to adhere to traumatized or oozing surfaces. Polyester/ collagen and methylene blue can adhere to oozing surfaces, whilst it is unknown for the remaining barriers. N-isopropylacrylamide, C17 glycerin ester and dimethyl-sulfoxide can be applied laparoscopically, while this is not possible for polyester/ collagen, polyhydroxyethylmethacrylate and poly(lactic-co-glycolic acid)/ epigallocatechin-3-O-gallate (223,224,226–228) . It is unknown as to whether methylene blue or poly(lactic-co-glycolic acid)/ epigallocatechin-3-O-gallate can be applied laparoscopically. Polyester/ collagen has a poor level of safety reported in animal studies (144,210), with unknown level of the ease of barrier application. N-isopropylacrylamide and dimethyl-sulfoxide have high level of reported ease of application, but unknown level of safety for the patient. Patient safety and ease of application are unknown for the remaining barriers. Characteristics of cost cost-effectiveness and post-operative pain are unknown for all the barriers in the group.

## Appendix S7 - Characteristics of barrier agents

| **Barrier Type** | | **Adherent to traumatized surface (Yes/No/Liquid)** | **Adherent to oozing surface (Yes/No/*)** | **Applicable through laparoscope (Yes/No/*)** | **Safe for patient (Likert scale*)** | **Cost-effective (Likert scale*)** | **Post-operative pain (Likert scale*)** | **Easiness of application (Likert scale*)** |
| --- | --- | --- | --- | --- | --- | --- | --- | --- |
| Algae | |  |  |  |  |  |  |  |
|  | Alginate | Yes | Yes | Yes |  |  |  |  |
|  | Agar films | Yes |  |  | 1 |  |  |  |
|  | Alginate/ Hyaluronic Acid | Yes | Yes | Yes |  |  |  |  |
| Cellulose | |  |  |  |  |  |  |  |
|  | Oxidised regenerated cellulose | Yes | No | Yes | 5 | On the market | 5 | 3 |
|  | Modified xyloglucan hydrogel | Yes |  | Yes | 5 |  | 5 | 5 |
|  | Carboxymethylcellulose | No | No | Yes |  |  |  |  |
|  | Carboxymethylcellulose/ Hyaluronic Acid | Yes | Yes | Yes | 5 |  | 5 | 5 |
|  | Carboxymethylcellulose/ Polyethylene Glycol | No | No | Yes |  |  |  | 4 |
| Chitosan | |  |  |  |  |  |  |  |
|  | N,O-carboxymethyl chitosan | Yes | Yes |  | 5 | 5 | 3 | 5 |
|  | Hydroxybutyl Chitosan | Yes | Yes | Yes |  |  |  |  |
|  | Chitosan / Carboxymethylcellulose /collagen | Yes | Yes |  |  |  |  |  |
|  | N,O-carboxymethyl chitosan / Hyaluronic Acid | No |  | Yes |  |  |  | 5 |
|  | Chitosan / gelatin | No | No | Yes |  |  |  |  |
|  | N,O-carboxymethyl chitosan / Dextran | Yes |  | Yes |  |  |  |  |
|  | Chitosan / Polyglycolic Acid | Yes |  |  |  |  |  |  |
| Glycoprotein | |  |  |  |  |  |  |  |
|  | Fibronectin derivative | Yes |  |  |  |  |  |  |
|  | Lactoferrin | No | No | Yes |  |  |  |  |
|  | Fibrin | Yes | Yes |  |  |  |  |  |
|  | Gelatin/Polyglycan ester | Yes | Yes | Yes | 5 | 5 |  |  |
|  | Gelatin/proteoglycan | No | No | Yes |  |  |  |  |
| Hyaluronic Acid | |  |  |  |  |  |  |  |
|  | Hyaluronic acid Hydrogel | Yes | Yes | Yes | 4 |  | 5 | 5 |
|  | Crosslinked Hyaluronic Acid | No | No | Yes | 4 | 4 | 5 | 4 |
|  | Hyaluronic Acid membrane | No |  | No | 5 |  |  | 4 |
| Icodextrin | |  |  |  |  |  |  |  |
|  | Icodextrin | No | No | Yes | 4 | 4 | 4 | 4 |
| Miscellaneous | |  |  |  |  |  |  |  |
|  | Dextran 70 | Yes | Yes | Yes |  |  |  |  |
|  | Phosphorylcholine | No | No | Yes |  |  |  | 4 |
|  | Silk | No | No |  |  |  |  | 3 |
|  | Ancrod | Yes | Yes | Yes |  |  |  | 4 |
|  | Bromelain | No |  | Yes |  |  |  | 5 |
|  | Xanthan gum | Yes |  | Yes |  |  |  | 5 |
|  | Pectin | Yes |  | Yes |  |  |  |  |
|  | Modified pullulan | Yes | Yes | Yes |  |  |  |  |
|  | Liquid paraffin | No | No | Yes |  |  |  |  |
|  | Galls ethyl acetate | Yes | Yes | Yes |  |  |  | 4 |
|  | Ethyl pyruvate | No | No | Yes |  |  |  | 5 |
|  | Tongfu Xiere Enteroclysis Mixture | No |  | Yes |  | 5 |  | 5 |
| Starch | |  |  |  |  |  |  |  |
|  | Sterile hydrophilic starch | Yes | Yes | Yes | 5 |  | 4 | 5 |
|  | Dextrin | Yes | Yes | Yes |  |  |  |  |
| Polycaprolactone | |  |  |  |  |  |  |  |
|  | Polycaprolactone / Polyhydroxybutyrate | No | No | Yes |  |  |  |  |
|  | Polycaprolactone / Hyaluronic Acid | Yes |  |  |  |  |  |  |
|  | Polycaprolactone / Polyethylene Glycol | Yes | Yes | Yes |  |  |  |  |
|  | Polycaprolactone / Gelatin | No | No |  |  |  |  |  |
| Polyethylene Glycol | |  |  |  |  |  |  |  |
|  | Polyethylene Glycol | No | No | Yes | 5 | 4 | 4 | 5 |
|  | Poloxamer 407 | Yes |  | Yes |  |  |  | 5 |
|  | Poloxamer 407/alginate | Yes |  | Yes | 5 |  |  | 5 |
| Polyglycolic Acid | |  |  |  |  |  |  |  |
|  | Polyglycolic Acid | No | No |  |  |  |  | 3 |
|  | Polylactic Acid | No | No | No |  |  |  | 4 |
|  | Polylactic Acid / Polyethylene Glycol | Yes | No |  | 5 |  | 3 | 3 |
|  | Polylactic Acid / Polycaprolactone | Yes |  | No |  |  |  | 4 |
|  | Poly(l-lactic acid) / modified mesoporous silica/ Ibuprofen | Yes | Yes | No |  |  |  |  |
| Polypropylene | |  |  |  |  |  |  |  |
|  | Polypropylene | No | No |  |  |  |  |  |
|  | Polypropylene/ glycolide/ Polycaprolactone | No | No | Yes | 2 |  |  |  |
|  | Polydioxanone/polypropylene/ Carboxymethylcellulose | No | No | Yes | 5 |  | 5 | 5 |
|  | Polypropylene/Titanium | No | No |  |  |  |  |  |
|  | Polypropylene/Omega 3 | No | No |  |  |  |  |  |
| Polyvinyl Alcohol | |  |  |  |  |  |  |  |
|  | Polyvinyl Alcohol hydrogel | Yes |  |  |  |  |  |  |
|  | Polyvinyl Alcohol / Carboxymethylcellulose | Yes | Yes | Yes, Gel |  |  |  |  |
| Silicone | |  |  |  |  |  |  |  |
|  | Polysiloxane | Yes |  |  |  |  |  | 5 |
|  | PolYesterurethane/polydimethylsiloxane | No | No |  |  |  |  |  |
| Miscellaneous | |  |  |  |  |  |  |  |
|  | Chitosan/Poly(d,l-lactic-co-glycolic acid)/Polyethylene oxide | Yes |  |  |  |  |  |  |
|  | PolYester/collagen | Yes | Yes | No | 4 |  |  |  |
|  | N-isopropylacrylamide | No | No | Yes, Hydrogel |  |  |  | 4 |
|  | C17 glycerin ester | Yes |  | Yes, Liquid |  |  |  |  |
|  | Methyline blue | Yes | Yes |  |  |  |  |  |
|  | Dimethyl-sulfoxide | No | No | Yes, Liquid |  |  |  | 5 |
|  | Polyhydroxyethylmethacrylate | Yes |  | No |  |  |  |  |
|  | Poly(lactic-co-glycolic acid) /Epigallocatechin-3-O-gallate | No | No | No |  |  |  |  |
